# 

**DOI:** 10.1017/psy.2025.10048

**Published:** 2025-10-06

**Authors:** Jordan Rickles

**Affiliations:** School of Education and Information Studies, University of California, Los Angeles, Los Angeles, CA, USA

## 1 Introduction

At its core, causal inference is concerned with how we know when an observed relationship between two events indicates that one event caused the other. Or to put a spin on a common aphorism, under what conditions are we comfortable inferring that correlation *does* imply causation? The desire to explain the cause(s) of an event or understand the effect of a cause can be traced from creation myth storytelling and ancient philosophy to central thinkers defining the Renaissance and Scientific Revolution. Given the fundamental role of causal reasoning over the history of human existence, the formalization of causal inference as an area of study within academic fields is relatively young. Modern causal inference draws from formative thinking during the 1920s on randomized experiments (Fisher, 1925; Neyman, 1923), path analysis (Wright, 1921), and instrumental variables (Wright, 1928), as well as seminal work during the middle of the twentieth century on nonexperimental studies (e.g., Cochran, 1953; Cochran & Chambers, 1965) and the validity of study findings (Campbell, 1957; Campbell & Stanley, 1963).

Three causal frameworks shape current causal inference methodology (Steiner et al., 2023): (a) the validity typology framework, (b) the potential outcomes framework, and (c) structural causal models. Based on the work of Campbell, Cook, and Shadish (Shadish et al., 2002), the validity typology is perhaps the most familiar framework in psychology. The framework uses four types of validity and their respective validity threats to evaluate whether conclusions from an analysis can be interpreted as causal or not, and can be generalized beyond the study sample. Originating from statistics (Holland, 1986; Rubin, 1974, 2005), the potential outcomes framework established an explicit statistical definition of a causal effect and formalized the assumptions required for unbiased estimation of causal effects. With roots in path analysis, simultaneous equations, and structural equation models, Pearl (1998, 2009) established a framework for causal inference based on structural causal models and causal graphs. The structural causal model framework established formal graphical criteria for the identification of causal effects, given the explicit articulation of the assumed data-generating process via a causal graph. In their excellent review of all three frameworks, Steiner et al. (2023) provided the following summary of the three approaches to causal inference: the validity typology is a practitioner’s guide to causal inference, the potential outcomes framework is a statistician’s guide to causal inference, and the structural causal model framework is a theoretician’s guide to causal inference.

**Figure 1:**
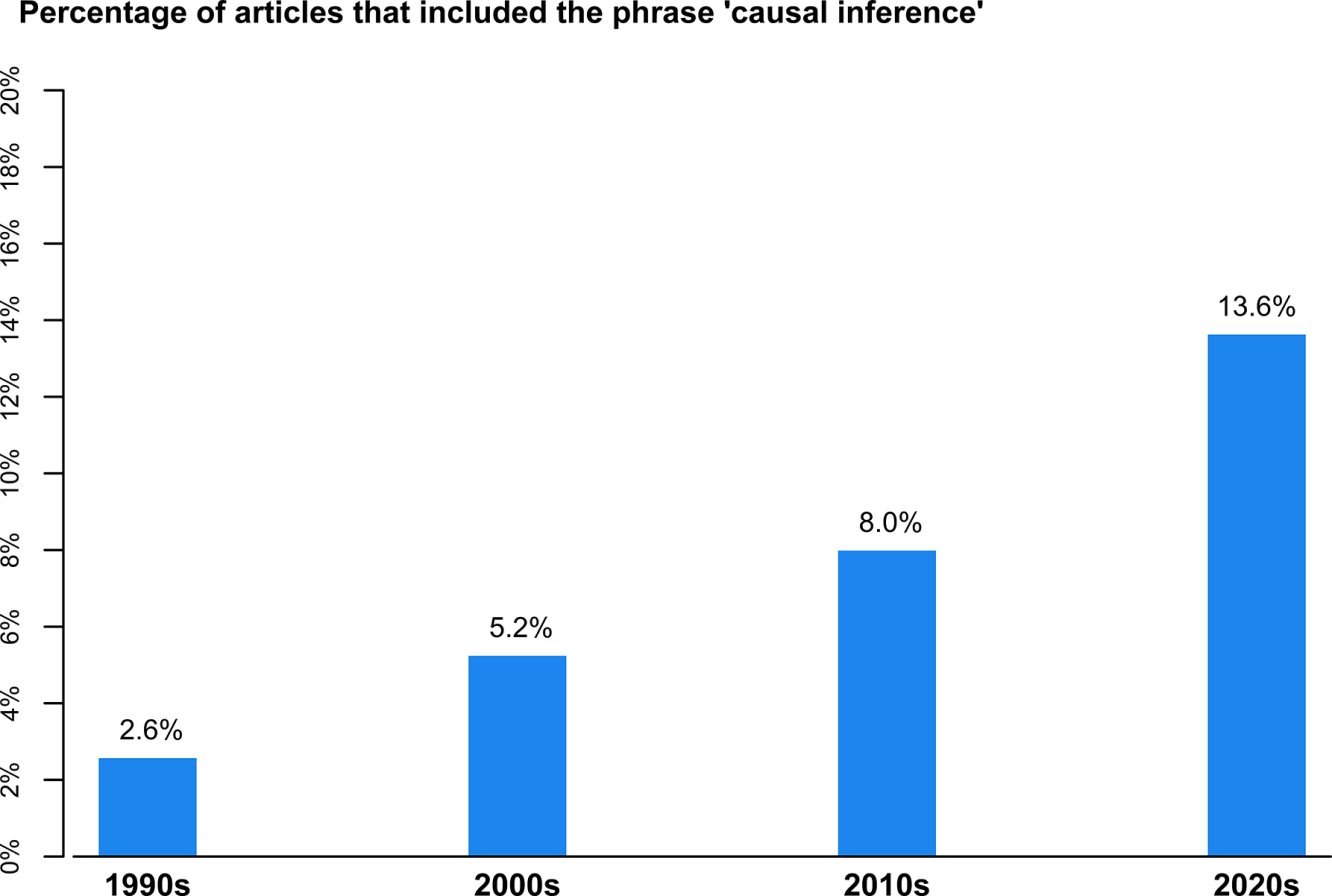
Percentage of Google Scholar search results for journal articles that included the phrase “causal inference,” by decade. *Note*: The Google Scholar search was conducted on June 16, 2025, and was restricted to the following four journals: *Journal of Educational and Behavioral Statistics*, *Multivariate Behavioral Research*, *Psychological Methods*, and *Psychometrika*.

Despite its historical connection to causal reasoning and methodological advances throughout the twentieth century, the wide acceptance and expansion of causal inference in statistics and the social sciences happened over the past 25 years. Pearl and Mackenzie (2018) labeled this recent period the “Causal Revolution.” For example, in four prominent journals for psychometricians and related methodologists, the percentage of articles mentioning causal inference increased fivefold from the 1990s to the 2020s (see Figure 1). In addition, 15 academic books on causal inference (by my count) were published between 2007 and 2024. A count that does not include books focused on specific causal inference methods, such as propensity score matching (e.g., Guo & Fraser, 2014), or more general books on statistical methods that may include substantive sections on causal inference (e.g., Gelman et al., 2021). *A First Course in Causal Inference* by Peng Ding, the focus of this review, is the latest causal inference textbook published during this period.

## 2 Book overview and content

Ding makes it clear from the start that *A First Course in Causal Inference* “covers causal inference from the statistics, biostatistics, and econometrics perspective” (p. xxi). Reinforcing Steiner et al.’s (2023) characterization of the potential outcomes framework as a statistician’s guide to causal inference, the potential outcomes framework is the central orientation throughout the book and the connective tissue across the book’s 29 chapters. The chapters are organized into six parts that are logically ordered to guide readers from foundational concepts to more complicated applications, with the core parts of the book organized around different types of treatment assignment mechanisms. Throughout, the topic of a given chapter is covered through a combination of statistical definitions, proofs, estimation methods, and applied or simulated examples with supporting R code. Each chapter includes sample homework problems at the end.

The first two chapters of the book comprise Part I, introducing important statistical concepts and the potential outcomes framework. This brief introduction to the foundational content is supported by a substantive appendix (technically Part VII) that reviews the probability and statistics concepts readers should be familiar with before progressing through the main text. Part II of the book focuses on randomized experimental designs. In a departure from many contemporary textbooks on causal inference, *A First Course in Causal Inference* allocates more space (almost 100 pages across seven chapters) to the effect estimation for randomized experiments than to any other topic covered in the book. Ding acknowledges that this is an unconventional choice and suggests that some may want to focus on a subset of the content in Part II. I appreciated the extended focus on randomized experiments. It helps establish an understanding of potential outcomes and important causal inference concepts before transitioning to nonexperimental designs. However, the detailed discussion of Fisherian inference, Neymanian inference, and rerandomization felt overly technical and nuanced for everyone besides aspiring statisticians. To facilitate differentiation of topics in Part II for those more interested in the design than the statistics, Part II may have worked better if it were separated into two sections: one focused on the conceptual importance and design of randomized experiments, and the other section focused on the estimation of effects.

Part III focuses on the estimation of causal effects in nonexperimental, or observational, studies. The six chapters in this section cover identification assumptions in nonexperimental studies, propensity score estimation, weighting, and matching. As with Part II, the chapter content emphasizes statistical definitions and estimation of causal effects more so than the practical rationale for different methodological approaches applied researchers will have to make when designing and executing a nonexperimental study. The important concepts and underlying approaches are well covered, but most students and applied researchers interested in these methods will need to seek out additional readings for a more comprehensive understanding of specific nonexperimental methods.

In Part IV, the book addresses situations where the identification assumptions required for unbiased effect estimation in nonexperimental studies might break down. Topics in this section cover assessing the conditional ignorability assumption (unconfounded assignment), sensitivity analysis, and regression discontinuity design (RDD). Ding takes an unconventional approach to RDD, framing it as a way to address a lack of covariate overlap rather than introducing RDD as a standalone quasi-experimental research design. Other causal inference textbooks, particularly those drawing from economics (e.g., Cunningham, 2021; Huntington-Klein, 2021; Murnane & Willett, 2010) or the validity framework (Shadish et al., 2002), give more attention to RDD as an important design option for causal inference. Part IV also marks the point in the book where Ding introduces the structural causal model, or at least the directed acyclic graph (DAG). The inclusion of DAGs is a welcome addition to the book and useful for illustrating important concepts. Their sudden appearance midway through the book and limited use outside a few chapters, however, reiterate that the potential outcomes framework remains the book’s focal paradigm. While structural causal models and DAGs play a more prominent role in many contemporary causal inference books (e.g., Brumback, 2021; Cunningham, 2021; Hernán & Robins, 2020; Huntington-Klein, 2021; Morgan & Winship, 2014), *A First Course in Causal Inference* uses DAGs sparingly and does not present structural causal models as any substantive tool for causal inference.

Part V focuses on instrumental variables, covering the method’s use for encouragement designs, noncompliance, fuzzy RDD, and Mendelian randomization. The chapters on instrumental variables take on a more applied and practical flavor than many of the book’s previous chapters. Emphasis is placed on the reason for an instrumental variables approach (e.g., to address noncompliance and estimate the causal average complier effect), the assumptions required for an unbiased effect estimate, and the demonstration of the method. Many of the technical details regarding theorems and proofs are relegated to the sample homework problems.

In the preface, Ding describes Part VI as a discussion of “special topics” that are “optional in some sense” (p. xviii). While it may be challenging to address the topics covered in Part VI in a single semester course designated as a “first course in causal inference,” the preface description undersells the cohesion, as well as the importance, of the four chapters in this final section of the book. The chapters in Part VI address situations where one may need to, or want to, condition on a post-treatment variable, which includes principal stratification, mediation analysis, and studies with time-varying treatments. The chapters on mediation analysis and time-varying treatments may be particularly salient to psychology students.

## 3 Discussion

*A First Course in Causal Inference* provides a quality introduction to, and at times an in-depth presentation of, most topics one would want from a “first” causal inference course. It does so through the lens of the potential outcomes framework and with (prospective) statisticians and related quantitative methodologists as the focal audience. This framing is understandable, given that the book is based on Ding’s years teaching a causal inference statistics course for undergraduate and graduate students and draws a lot of inspiration from the Imbens and Rubin (2015) causal inference book. The focus and framing are not a criticism of the book’s value. Ding has provided the field with a quality resource for understanding causal inference and the statistics needed to support the estimation of causal effects. The discussions, simulations, and applications facilitate comprehension of an array of methods for causal inference, and the supporting R code is a nice bonus.

I recommend the book as the primary text for a statistics-focused graduate-level course. The breadth of topics and applications covered makes it relevant not just for students in statistics but also for students in other statistics-adjacent programs, such as psychometrics, data science, biostatistics, and epidemiology. The book’s emphasis on statistical definitions and equations means it is not as appropriate for a graduate-level methods course intended for a more applied audience or undergraduate students. While Ding uses applied examples throughout—mostly drawing from classic examples in health, education, and economics research—and includes R code to demonstrate application of the statistics, they are not presented or supported with applied researchers in psychology or education as the primary audience. The book, however, is a good resource for instructors of any course focused on causal inference, as well as researchers looking to reinforce their understanding of causal inference from a statistical perspective.

With many other causal inference textbooks now available, it is important to consider what *A First Course in Causal Inference* is not. The book, for example, makes no connection to the validity typology that may be more familiar in psychology and more salient for applied researchers. Similarly, many of the chapters emphasize statistical definitions, estimation formulas, and proofs over a discussion of the conceptual rationale behind different methods and the design considerations applied research will face in field-based applications. Books by Morgan and Winship (2014), Murnane and Willett (2010), and Shadish et al. (2002) may be better options for a more applied and less technical take on causal inference. When it comes to coverage of causal inference methods and designs, Ding is upfront in the preface about excluding methods like difference-in-difference and synthetic control methods, as well as more advanced topics like the use of machine learning and Bayesian methods for causal inference. In addition, experimental designs such as factorial designs and sequential multiple assignment randomized trials are not discussed, and the book does not address causal inference with multilevel, or clustered, data. Absent, too, is any discussion of how missing data and measurement error influence our ability to draw valid causal inferences from the observed data.

One should not, however, expect any one textbook to cover all possible designs and methods for causal inference, nor all data complications that arise in applied studies. The breadth and depth of the causal inference literature are a testament to the foundational work of the twentieth century and how much the field has grown during this current “Causal Revolution” era. *A First Course in Causal Inference* is both a positive effect of this revolution and a potential cause of continued growth and future methodological advancements.

## References

[r1] Brumback, B. A. (2021). Fundamentals of causal inference: With R. Chapman and Hall/CRC.

[r2] Campbell, D. T. (1957). Factors relevant to the validity of experiments in social settings. Psychological Bulletin, 54(4), 297–312. 13465924 10.1037/h0040950

[r3] Campbell, D. T. , & Stanley, J. C. (1963). Experimental and quasi-experimental designs for research. Rand McNally.

[r4] Cochran, W. G. (1953). Matching in analytical studies. American Journal of Public Health, 43, 684–691. 13040588 10.2105/ajph.43.6_pt_1.684 PMC1620280

[r5] Cochran, W. G. , & Chambers, S. P. (1965). The planning of observational studies of human populations. Journal of the Royal Statistical Society. Series A (General), 128(2), 234–266.

[r6] Cunningham, S. (2021). Causal inference: The mixtape. Yale University Press.

[r7] Fisher, R. A. (1925). Statistical methods for research workers. Oliver and Boyd.

[r8] Gelman, A. , Hill, J. , & Vehtari, A. (2021). Regression and other stories. Cambridge University Press.

[r9] Guo, S. , & Fraser, M. W. (2014). Propensity score analysis: Statistical methods and applications. SAGE Publications.

[r10] Hernán, M. A. , & Robins, J. M. (2020). Causal inference: What if. Chapman & Hall/CRC.

[r11] Holland, P. W. (1986). Statistics and causal inference. Journal of the American Statistical Association, 81(396), 945–960.

[r12] Huntington-Klein, N. (2021). The effect: An introduction to research design and causality. Chapman and Hall/CRC.

[r13] Imbens, G. W. , & Rubin, D. B. (2015). Causal inference in statistics, social, and biomedical sciences. Cambridge University Press.

[r14] Morgan, S. L. , & Winship, C. (2014). Counterfactuals and causal inference: Methods and principles for social research. Cambridge University Press.

[r25] Murnane, R. J. , & Willett, J. B. (2010). Methods Matter: Improving Causal Inference in Educational and Social Science Research. Oxford University Press.

[r15] Neyman, J. (1923). On the application of probability theory to agricultural experiments: Essay on principles. Section 9. Statistical Science, 5(4), 465–480.

[r16] Pearl, J. (1998). Graphs, causality, and structural equation models. Sociological Methods & Research, 27(2), 226–284. 10.1177/0049124198027002004

[r17] Pearl, J. (2009). Causality. Cambridge University Press.

[r18] Pearl, J. , & Mackenzie, D. (2018). The Book of Why: The New Science of Cause and Effect. Basic Books.

[r19] Rubin, D. B. (1974). Estimating causal effects of treatments in randomized and nonrandomized studies. Journal of Educational Psychology, 66(5), 688–701.

[r20] Rubin, D. B. (2005). Causal inference using potential outcomes: Design, modeling, decisions. Journal of the American Statistical Association, 100(469), 322–331.

[r21] Shadish, W. R. , Cook, T. D. , & Campbell, D. T. (2002). Experimental and quasi-experimental designs for generalized causal inference. Houghton, Mifflin and Company.

[r22] Steiner, P. M. , Shadish, W. R. , & Sullivan, K. J. (2023). Frameworks for causal inference in psychological science. In APA handbook of research methods in psychology: Foundations, planning, measures, and psychometrics (Vol. 1, 2nd ed., pp. 23–56). American Psychological Association.

[r23] Wright, P. G. (1928). The tariff on animal and vegetable oils. Macmillan.

[r24] Wright, S. (1921). Correlation and causation. Journal of Agricultural Research, 20(7), 557–585.

